# The Dangers of Chest Compression: A Case Report

**DOI:** 10.7759/cureus.116582

**Published:** 2026-09-20

**Authors:** A Abdul Hai, Graeme Currie, David Miller, Mohammed Khalil

**Affiliations:** 1 Respiratory Medicine, Aberdeen Royal Infirmary, NHS Grampian, Aberdeen, GBR; 2 Cardiothoracic Surgery, Aberdeen Royal Infirmary, NHS Grampian, Aberdeen, GBR

**Keywords:** bystander cpr, cardiopulmonary resuscitation, complications (cpr), out-of-hospital cpr, post-cpr complication, traumatic cpr

## Abstract

Chest injuries after cardiopulmonary resuscitation (CPR) are common, but subsequent infected parasternal collections after CPR are uncommonly described. We present a case of a 49-year-old female with a history of intravenous drug use (IVDU) who developed multiple sternal and rib fractures and a large infected parasternal collection after community CPR following a heroin overdose. She was treated with antibiotics and required surgical evacuation of the collection. This case highlights the importance of recognizing and managing post-CPR chest wall complications early to prevent serious morbidity.

## Introduction

Chest wall injuries are well-recognized complications of cardiopulmonary resuscitation (CPR). These include rib and sternal fractures, haematomas, and rarely, infection of the chest wall collections. Although traumatic chest wall haematomas are relatively common after CPR, secondary infection with abscess formation and sepsis is exceedingly rare [1]. This report describes an infected parasternal collection following CPR-related chest wall trauma with associated sternal and rib fractures, successfully managed with antibiotics and surgical drainage.

## Case presentation

A 49-year-old female presented with severe chest pain and increasing breathlessness two weeks after an episode of CPR performed in the community. She had become unresponsive after injecting heroin, and community chest compressions continued until she regained consciousness. Since then, she has experienced persistent chest pain, a productive cough, and increasing swelling over the anterior chest.

Her past medical history included intravenous drug use (IVDU), a previous splenectomy after trauma, thoracic aortic dissection, and pulmonary embolism treated with rivaroxaban (direct oral anticoagulant). Initial blood investigations are summarized in Table 1.

**Table 1:** Laboratory investigations on admission CRP: C-reactive protein; PT: prothrombin time; INR: international normalised ratio; APTT: activated partial thromboplastin time

Investigation	Result	Reference range
Haemoglobin	94 g/L	115–160 g/L
White cell count	43.8 ×10⁹/L	4.0–11.0 ×10⁹/L
Platelet count	715 ×10⁹/L	150–400 ×10⁹/L
CRP	391 mg/L	<5 mg/L
Albumin	22 g/L	35–50 g/L
PT	16.9 s	10–13 s
INR	1.4	0.9–1.2
APTT	25.1 s	22–35 s

Computed tomography (CT) of the chest showed a displaced transverse sternal fracture with a large anterior parasternal collection (3 × 10 × 5 cm) containing gas locules, bilateral first to third rib fractures, and small pleural effusions (Figure 1).

**Figure 1:**
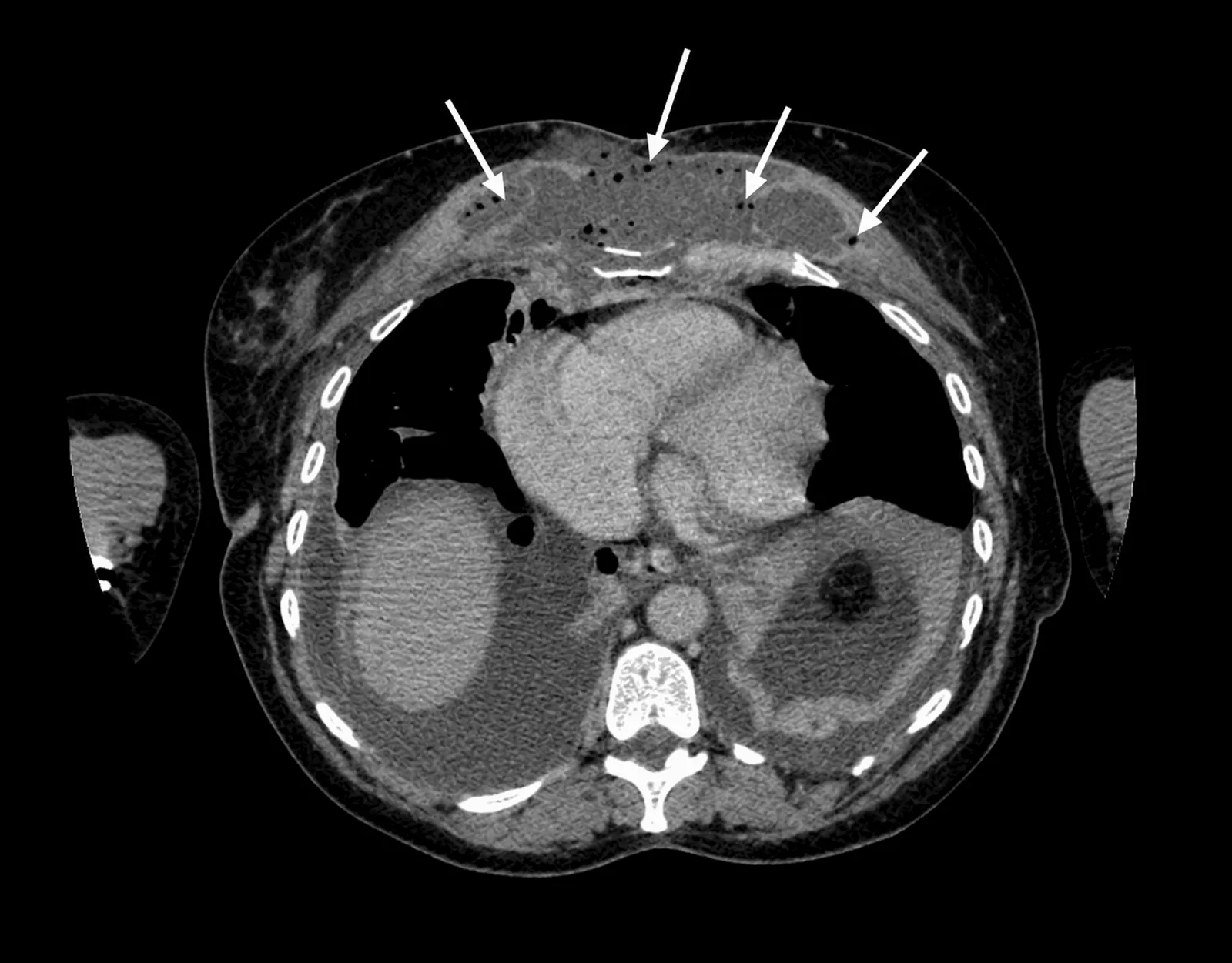
Contrast-enhanced CT scan showing a large anterior parasternal collection (white arrows) containing gas locules anterior to the fractured sternum.

Four days later, worsening pain and increasing anterior chest swelling prompted repeat CT, which showed an increased collection size (15 × 5 × 11 cm) with rupture into the subcutaneous space, consistent with progression of the chest wall collection. Clinical examination showed marked erythematous swelling over the anterior chest (Figure 2).

**Figure 2:**
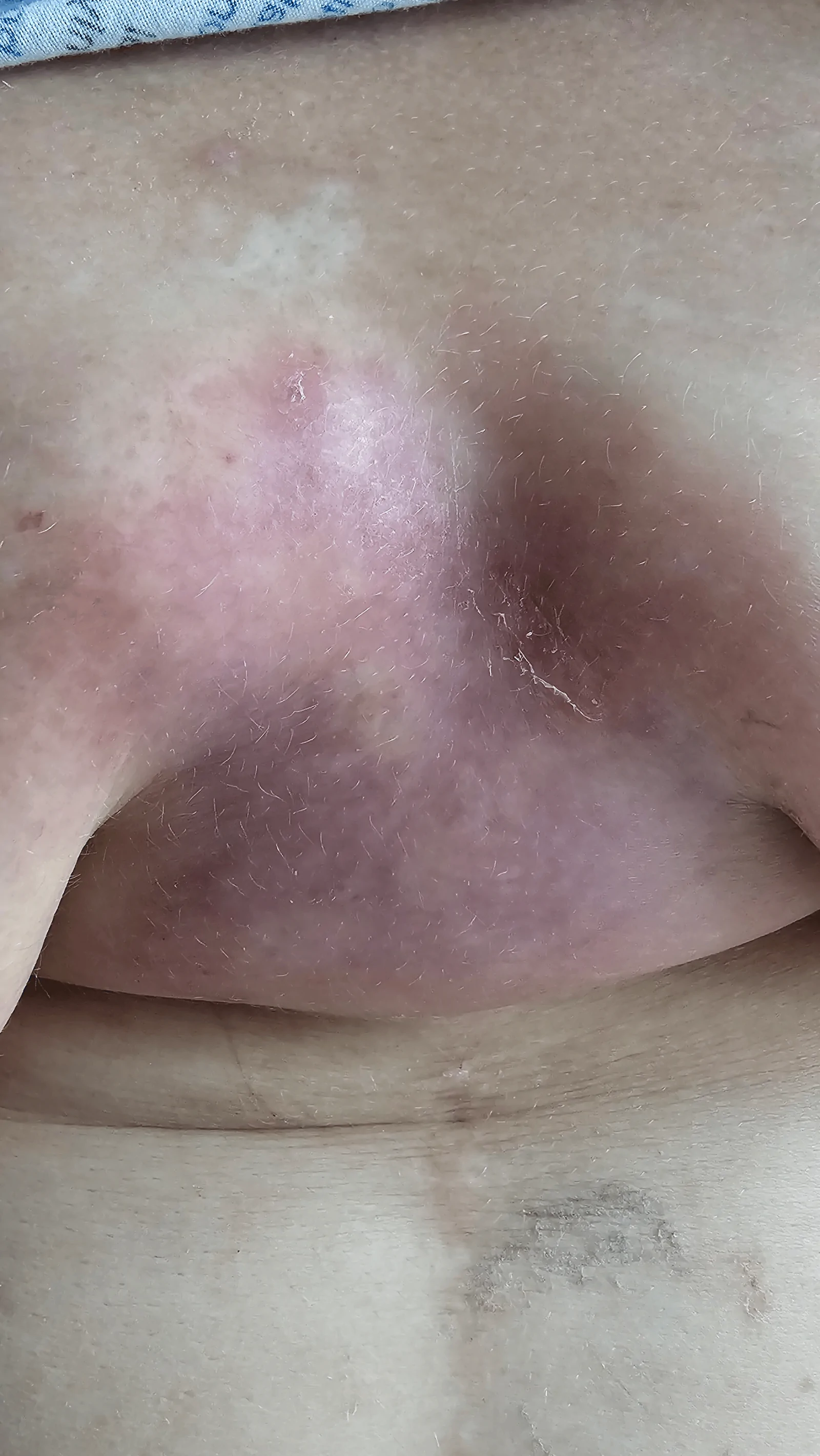
Anterior chest wall haematoma on physical examination.

Blood cultures grew *Staphylococcus aureus*, and subsequent echocardiogram showed normal systolic function without valvular vegetations.

She commenced on intravenous flucloxacillin, plus aztreonam and metronidazole. Pain was difficult to manage and required oral morphine and ketamine. Due to the increase in collection size, evacuation of the infected sternal collection was performed. A vacuum-assisted closure dressing was applied initially. Antibiotics were switched to oral flucloxacillin for a further two weeks. Following this, her inflammatory markers rose, and a further two weeks of co-trimoxazole were given. Her wound healed well, and she was discharged with outpatient follow-up for wound review.

## Discussion

Chest wall injuries are well-recognised complications of CPR, with sternal fractures reported in approximately 24% of cases and rib fractures in up to 55%. Parasternal or chest wall collections occur in around 12% of patients following CPR, typically arising from traumatic haematomas caused by chest compression. Although such collections are described in the literature, progression to deep chest wall infection or sepsis is far less frequently documented, making this case particularly important to highlight [1]. 

In this patient, the most plausible initial event was a parasternal haematoma secondary to chest compressions that subsequently became infected, likely with *Staphylococcus aureus*, introduced either through skin contamination or via IVDU. Once infection develops within a closed chest wall collection, it can rapidly extend into the mediastinum, posing a serious risk of life-threatening sepsis if not promptly identified and treated [2]. Early recognition is therefore important. Persistent chest pain, swelling, erythema, or systemic signs of infection following CPR should raise suspicion of an infected haematoma rather than attributing symptoms solely to rib or sternal fractures. CT plays a central role in early diagnosis, especially when there is clinical deterioration or enlarging collection [3-6]. Management involves appropriate antimicrobial therapy guided by microbiology and surgical drainage if the collection enlarges or fails to resolve. In this patient, the combination of IVDU, previous splenectomy (impaired immunity), anticoagulation, and community CPR (non-physician chest compressors) likely contributed to infection risk and delayed healing [2,3,7-10]. 

## Conclusions

This case shows that chest wall infections can occur after CPR, especially in patients with multiple comorbidities. Early imaging, antibiotic therapy, and surgical evacuation are key to successful recovery. Awareness of such rare complications can help prevent delays in diagnosis and reduce morbidity. 
